# Rising direct medical costs of osteoarthritis in Germany from 2002 to 2020: trends, demographic drivers, and public health considerations

**DOI:** 10.3389/fpubh.2025.1627510

**Published:** 2025-11-12

**Authors:** Hadrian Platzer, Simone Gantz, Berit Färber, Babak Moradi

**Affiliations:** 1 Department of Orthopedics and Trauma Surgery, University Medical Center Schleswig-Holstein, Campus Kiel, Kiel, Germany; 2 Orthopedic Research Center, Kiel University, Kiel, Germany; 3 Controlling Department, University Medical Center Schleswig-Holstein, Lübeck, Germany

**Keywords:** osteoarthritis, direct healthcare costs, cost-of-illness analysis, economic burden, aging, health policy, Germany

## Abstract

**Background:**

As a major chronic disease Osteoarthritis (OA) poses a substantial clinical and economic challenge, especially in aging societies. Worldwide the economic burden of OA is significant, however data on related healthcare costs in Germany remain limited. With mounting financial pressures, identifying key cost drivers in healthcare is becoming increasingly vital. This study offers a novel quantification of Germany’s direct medical OA costs, examining demographic trends, and exploring implications for healthcare planning and policy in an international context.

**Methods:**

Retrospective analysis of direct medical costs was conducted using data from the German Federal Statistical Office for the years 2002, 2004, 2006, 2008, 2015, and 2020. Costs were stratified by sex, age, and healthcare facility.

**Results:**

In 2020, diseases of the musculoskeletal system accounted for 10% of Germany’s direct healthcare costs, with osteoarthritis being one of the leading contributors to this economic burden. OA-related costs rose from €8.6 billion in 2015 to €12.1 billion in 2020 (+41%), particularly among older adults. While costs declined in those under 45, they increased by 17% in those aged 45–65, 32% in those aged 65–85, and 99% in those over 85. Inpatient and semi-inpatient costs rose by 32%, reaching €6.6 billion, driven by nursing care, which nearly doubled between 2015 and 2020. Outpatient OA costs totaled €3.4 billion in 2020, with outpatient nursing showing the sharpest rise (+85%). Gender-specific differences were substantial: women incurred 70% of total costs, with higher shares in nursing care, while men had relatively higher expenditures in hospital and rehabilitation settings.

**Conclusion:**

Osteoarthritis imposes a substantial and rapidly increasing economic burden on the German healthcare system, particularly due to adults aged 65 and older. Inpatient and nursing care have emerged as the primary cost drivers. In a European comparison, Germany ranks among the countries with the highest OA-related direct medical costs. These findings underscore the urgent need for osteoarthritis-specific public health strategies focused on prevention, individualized conservative treatments, and gender-sensitive care models to ensure the long-term sustainability of healthcare systems in aging societies.

## 1 Introduction

Healthcare systems around the world are facing substantial economic challenges due to competing priorities and rising demands on public funding. This situation necessitates comprehensive analysis and the development of targeted, evidence-based strategies. According to the Organisation for Economic Co-operation and Development (OECD), the United States spent 17.8% of its Gross Domestic Product (GDP) on healthcare expenditures in 2021, the highest among member countries – followed by Germany (1). In 2022, total healthcare expenditure in Germany reached €497.7 billion, corresponding to €5,939 per capita and representing 12.8% of GDP (2). Ensuring the financial sustainability and functional resilience of the healthcare system in the medium and long term requires strategic planning and burden-reducing interventions. In addition to cross-cutting, disease-independent reforms, condition-specific approaches may be warranted. Identifying high-cost diseases and analyzing their economic burden in detail can yield valuable insights for targeted policy design and resource allocation.

In Germany, demographic changes have led to a continuously aging population structure. Given the close association between age and Osteoarthritis (OA), a rise in OA-related healthcare needs and costs is inevitable. OA is the most common joint disease worldwide (3–5), leading not only to significant impairments in quality of life but also to substantial global healthcare costs (6, 7). However, the disease-related costs of OA in Germany remain insufficiently studied. In particular, a comprehensive analysis of the economic impact of OA over the course of the 21st century has not yet been conducted. Understanding the dynamics of direct healthcare costs associated with OA over time is crucial for planning and allocating healthcare resources efficiently.

By providing the first long-term analysis, this study aims to analyze the trends in direct medical costs attributable to OA in Germany over an extended time period (2002–2020), stratified by sex, age and healthcare sector. Additionally, the study seeks to discuss the public health implications of the observed trends and to provide insights relevant for future healthcare planning and policy-making.

## 2 Materials and methods

This study is based on publicly available national health expenditure data provided by the German Federal Statistical Office (Statistisches Bundesamt, DESTATIS) (8). The analysis includes cost data from the years 2002, 2004, 2006, 2008, 2015, and 2020. These specific years were selected based on the availability of most recent stratified disease cost reports (Krankheitskostenrechnungen) published by DESTATIS. DESTATIS applies a top-down cost-of-illness approach and allocates healthcare expenditures to specific diseases based on diagnostic codes from the International Statistical Classification of Diseases and Related Health Problems (ICD-10). The analysis of this study includes ICD-10 codes M15-M19, covering polyarthrosis (M15), hip OA (M16), knee OA (M17), rhizarthrosis (M18), and other or unspecified OA (M19). This coding structure ensures broad inclusion of osteoarthritis subtypes. However, potential coding inconsistencies – particularly in generalized or non-specific cases – represent an inherent limitation of secondary data use. This study is based on aggregated cost estimates and the data represent population-level healthcare expenditures stratified by diagnosis, sex, predefined age groups and healthcare sector. As these data are not derived from individual patient-level records, no measures of statistical dispersion (e.g., standard deviations, confidence intervals) or sample-based variability are available. Consequently, formal hypothesis testing (e.g., *p*-values) could not be conducted, and all results are presented descriptively. Cost values are reported in absolute euros for each year (2002, 2004, 2006, 2008, 2015, and 2020). All cost data are reported in nominal euros, as provided by DESTATIS in the national health accounts, without adjustment for inflation. This approach was chosen to reflect expenditure trends as officially reported and used for healthcare budgeting and policy-making in Germany.

The 2020 quality report highlights that differences in data sources and methodological approaches may limit the comparability of cost calculations over time (9). Since this study specifically analyzes the direct disease costs of osteoarthritis (ICD-10: M15-M19), a preliminary evaluation was conducted in collaboration with the Federal Statistical Office of Germany to determine which datasets were comparable across different time periods and to identify potential limitations and confounding factors. A gender- and age-specific comparison of direct osteoarthritis-related healthcare costs from 2002 to 2008 with those from 2015 and 2020 may be affected by methodological biases due to variations in data collection for ‘offices of physicians’ and ‘pharmacies,’ which accounted for approximately 30% of total expenditures. Indirect OA disease costs, such as lost work years due to temporary disability, permanent invalidity, or mortality, were not included in this study due to limited data availability. While such data were reported for osteoarthritis in the 2008 disease cost calculation, they were not included in the official reports for 2015 or 2020. Additionally, a direct statistical comparison analysis of osteoarthritis-related costs between countries was not performed due to substantial differences in data collection methodologies and healthcare systems. Data were analyzed descriptively, focusing on cost trends over time and subgroup differences. No ethical approval was required for this study, as only aggregated and anonymized data from public sources were used. Data analysis and graphical representations were conducted using GraphPad Prism (Version 9.3.1).

## 3 Results

The five diagnostic groups that contributed most significantly to direct disease costs in Germany in both 2015 and 2020 were C00-D48 ‘Neoplasms, ‘F00-F99 ‘Mental and Behavioral Disorders’, I00-I99 ‘Diseases of the Circulatory System’, K00-K93 ‘Diseases of the Digestive System’, and M00-M99 ‘Diseases of the Musculoskeletal System and Connective Tissue’. The latter accounted for 10% of total direct disease costs in Germany in 2020 (8).

### 3.1 Temporal trends in direct costs of musculoskeletal diseases (2002–2020)

**Figure 1:**
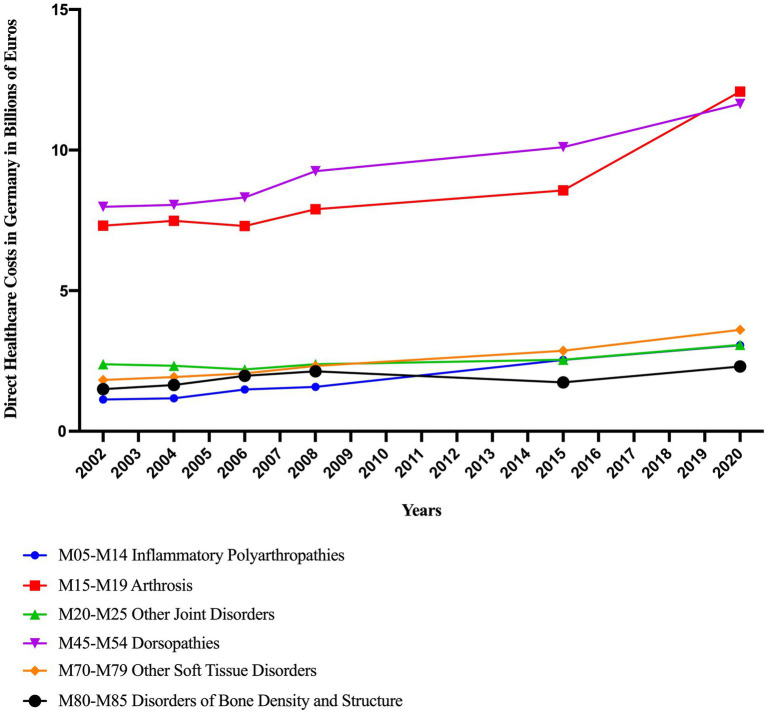
Direct disease costs associated with the ICD-10 diagnostic group ‘Diseases of the Musculoskeletal System and Connective Tissue’ in Germany from 2002 to 2020 (8).

The direct disease costs caused by disorders of the ‘Musculoskeletal System and Connective Tissue’ have increased substantially since 2002 (Figure 1). Within this diagnostic group, dorsopathies and osteoarthritis consistently ranked among the leading contributors to healthcare costs. In 2020, each of these two cost-intensive diagnoses accounted for more than €10 billion in direct expenses, whereas the other diagnostic groups remained below €5 billion. Osteoarthritis incurred the highest direct healthcare costs in 2020, rising from €8.6 billion in 2015 to €12.1 billion in 2020 – an increase of 41%.

### 3.2 Age- and sex-specific trends in OA-related costs over time

While costs remained largely stable and showed overall an absolute decline in age groups under 45 years, a marked increase was observed in the oldest age cohorts. Specifically, between 2015 and 2020, costs increased by 17% in the 45–65 age group, by 32% in the 65–85 age group, and by a striking 99% among individuals aged 85 years and older (8).

**Figure 2:**
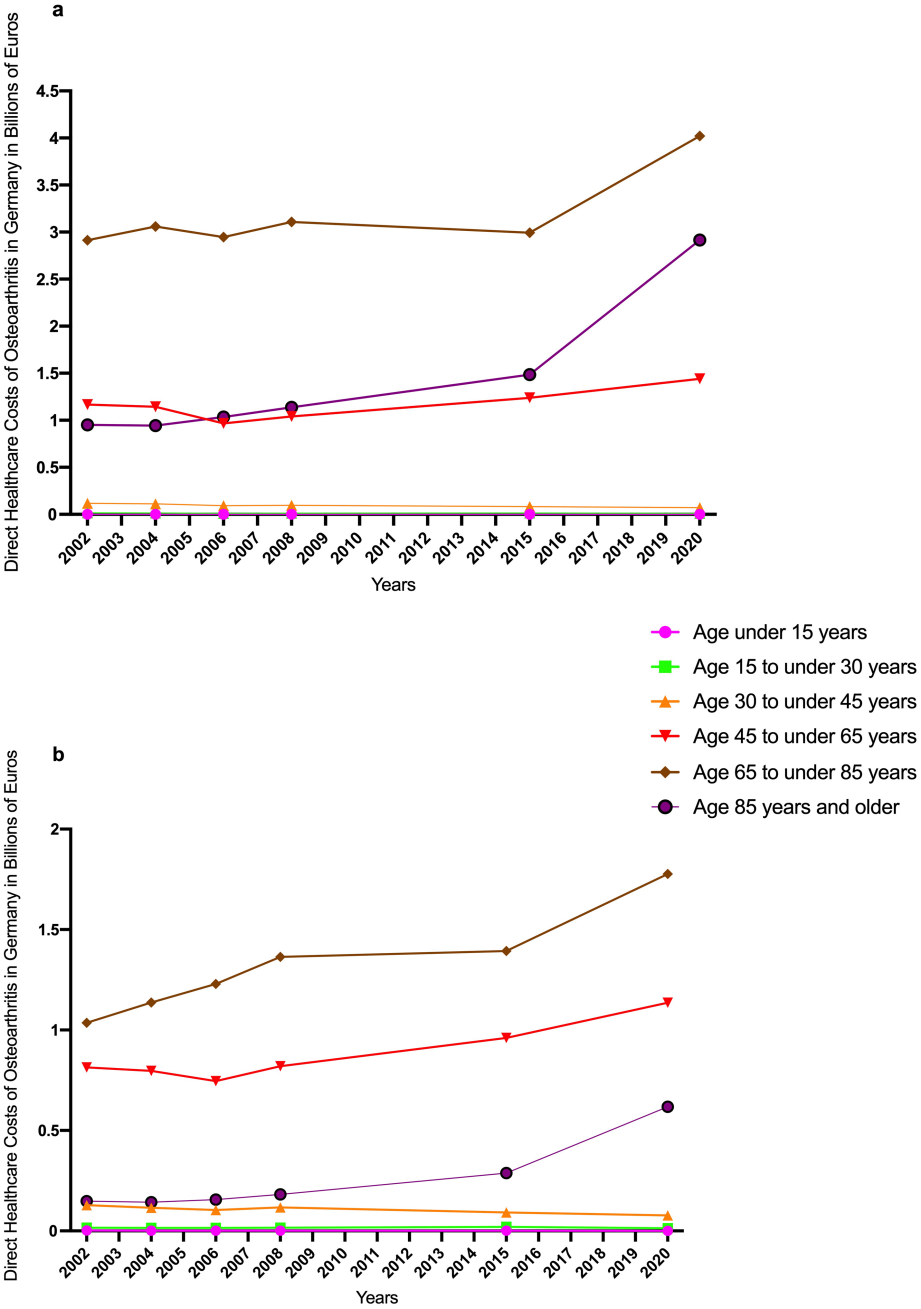
Trends in direct healthcare costs of osteoarthritis (ICD-10 M15-M19) in Germany from 2002 to 2020, stratified by sex and age group: women **(a)** and men **(b)** (8).

Figure 2 illustrates the progression of direct healthcare costs of OA associated with osteoarthritis from 2002 to 2020, stratified by age for both sexes: In patients aged 45 and older, costs rose for both sexes, with women continuously exceeding men. Among those aged 85 and older, costs increased from €952 million (women) and €148 million (men) in 2002 to €2.92 billion (women) and €618 million (men) in 2020. Gender differences in younger age groups (<45 years) were comparatively small and stable.

### 3.3 Temporal shifts in OA cost distribution across healthcare sectors

#### 3.3.1 Inpatient and semi-inpatient health care facilities

**Figure 3:**
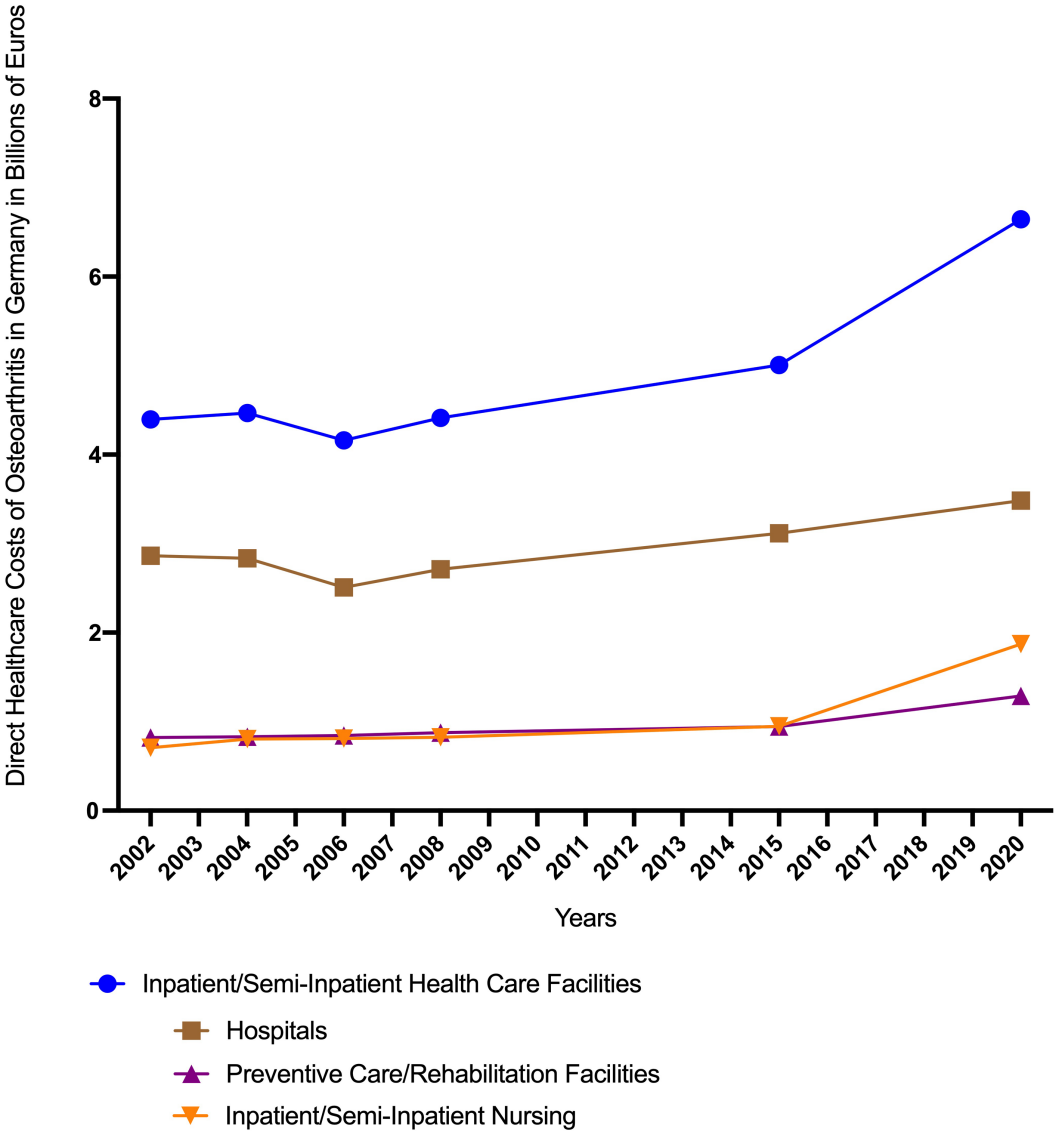
Direct healthcare costs of osteoarthritis in Germany attributable to inpatient and semi-inpatient healthcare facilities, categorized into ‘Hospitals’, ‘Preventive/Rehabilitation facilities’ and ‘Inpatient/semi-inpatient nursing’ (8).

The cost development in inpatient/semi-inpatient healthcare facilities is depicted in Figure 3, showing an increase in disease-related expenditures by 32%, from €5 billion in 2015 to €6.6 billion in 2020. All three analyzed categories – hospitals, preventive/rehabilitation facilities, and inpatient/semi-inpatient nursing care – exhibited a substantial rise in costs from 2002 to 2020. In 2020, hospitals accounted for the highest expenses, totaling €3.5 billion, which corresponded to 52% of the total direct osteoarthritis-related disease costs in inpatient/semi-inpatient healthcare settings. Particularly noteworthy is the sharp increase in expenditures for inpatient/semi-inpatient nursing care, which nearly doubled from €1 billion in 2015 to €1.9 billion in 2020 (see Figure 3).

#### 3.3.2 Outpatient health care facilities

**Figure 4:**
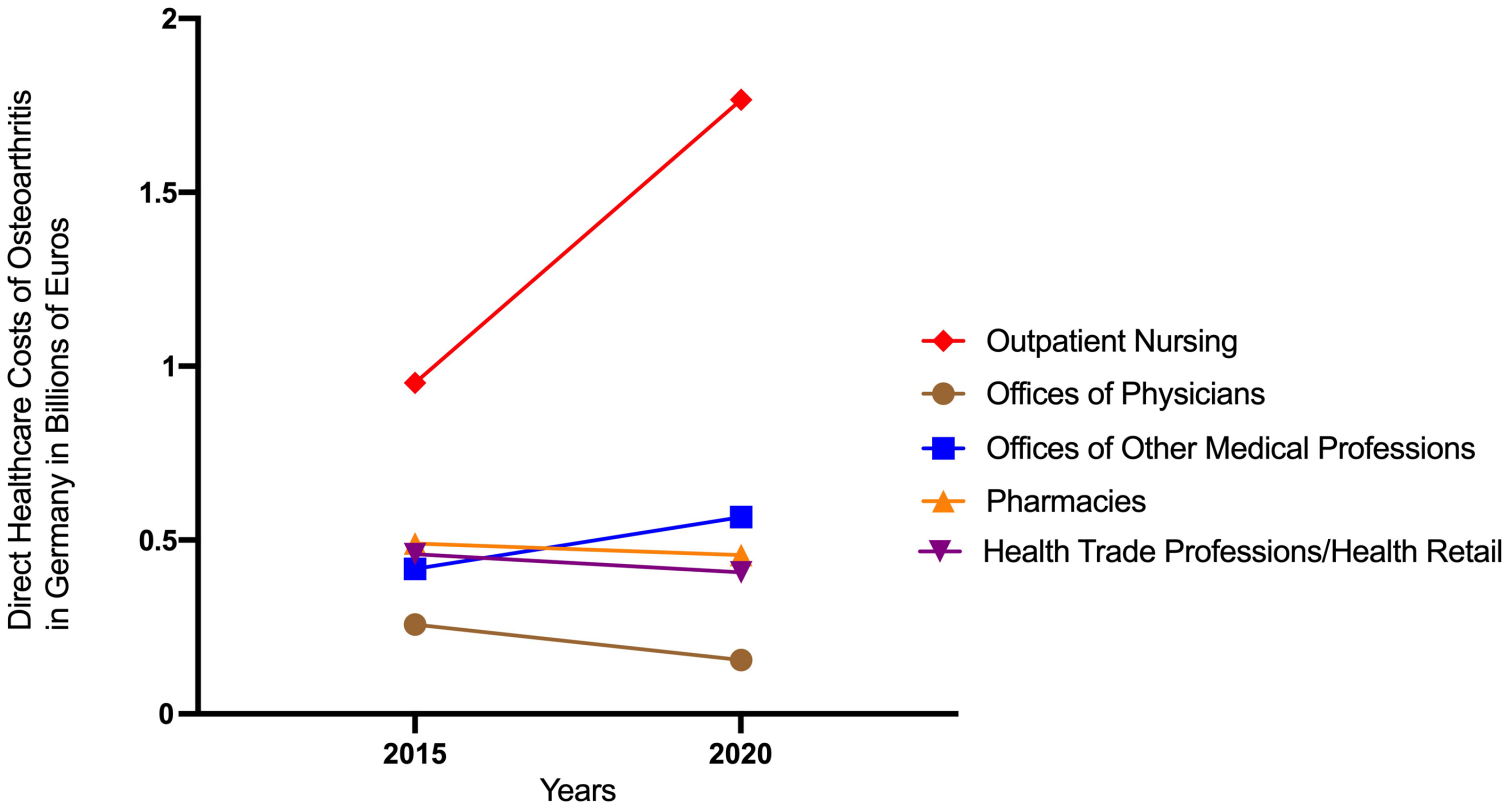
Direct healthcare costs of osteoarthritis in Germany attributable to outpatient care facilities in 2015 and 2020 (8).

In outpatient health care facilities direct OA costs reached €3.3 billion in 2020, representing a 30% increase compared to 2015 (see Figure 4). The sector-specific analysis reveals significant differences in cost development. The most pronounced increase was observed in outpatient nursing, where expenditures rose from €952 million in 2015 to €1.76 billion in 2020, accounting for 52.7% of total outpatient direct healthcare costs in that year. In contrast, expenditures for ‘offices of ther medical professions’ (e.g., physiotherapy, occupational therapy) showed more moderate growth, increasing from €417 million in 2015 to €566 million in 2020 (+36%). Conversely, costs declined in several areas, including ‘pharmacies’ (from €490 million to €457 million), ‘health trade professions/ health retail’ (from €460 million to €407 million) and ‘offices of physicians’ (from €257 million to €155 million).

### 3.4 Sex-specific OA cost distribution across care sectors in 2020

**Figure 5:**
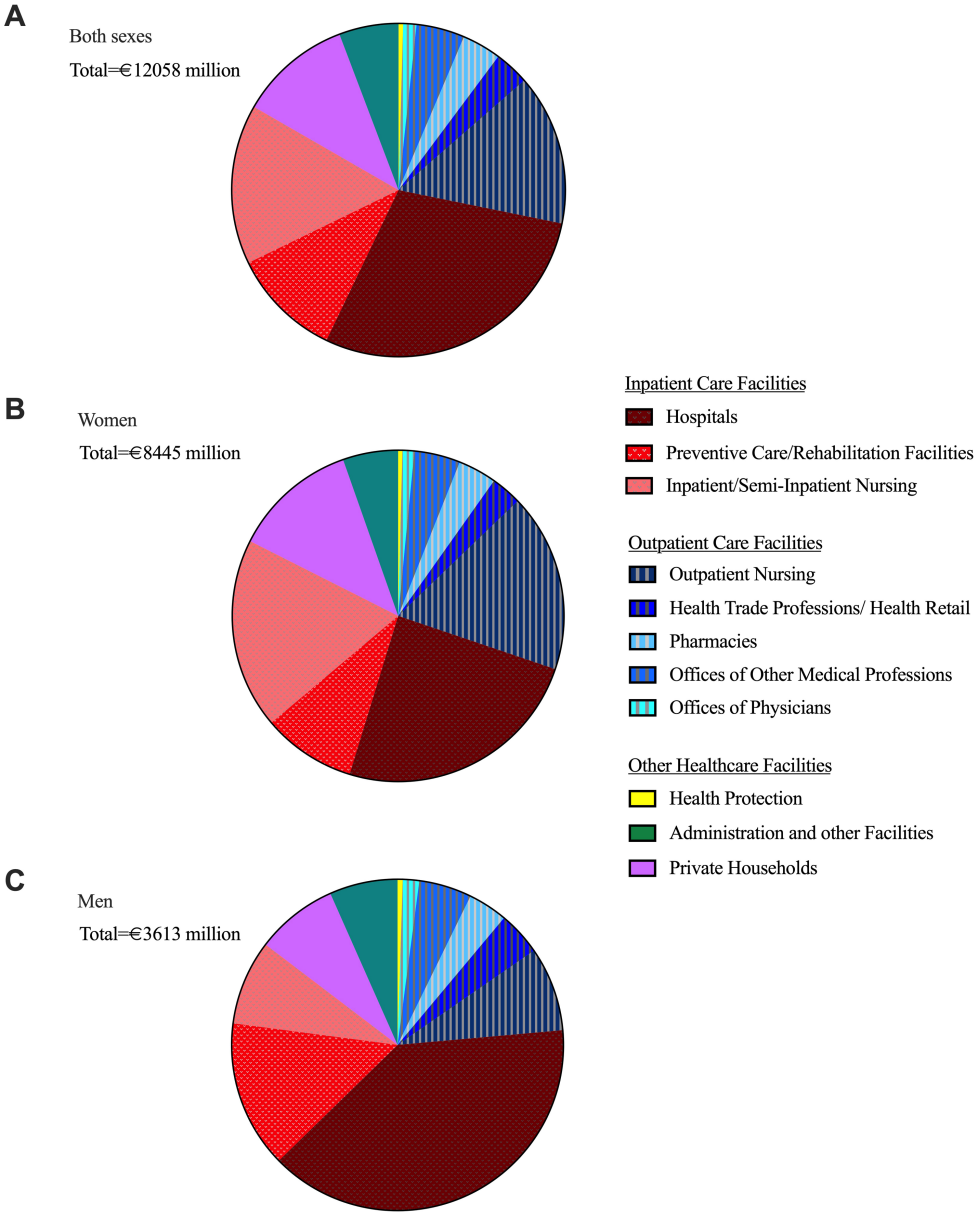
Relative proportion of direct healthcare costs of osteoarthritis in Germany (2020) by facility type (inpatient, outpatient care, and other healthcare facilities) in relation to total direct costs, overall **(a)** and by sex: women **(b)**, men **(c)** (8).

Of the total €12.1 billion in direct OA-related healthcare costs in 2020, 55% (€6.6 billion) were attributable to inpatient and semi-inpatient facilities, while 28% (€3.4 billion) were associated with outpatient care facilities (see Figure 5). Outpatient nursing services accounted for the largest share of outpatient costs at €1.8 billion. Consequently, expenditures for inpatient and semi-inpatient care were nearly twice as high as those for outpatient care. The combined costs of outpatient and inpatient/semi-inpatient nursing care service amounted to €3.6 billion, representing 30% of the total direct osteoarthritis-related healthcare expenditures.

Female patients accounted for 70% of the total costs (€8.4 billion), while male patients contributed for 30%. Relative to their respective total costs, men incurred significantly higher expenditures in hospitals (40% vs. 25% for women) and preventive care/rehabilitation facilities (14% vs. 9% for women). In contrast, women generated higher costs in outpatient nursing (17% vs. 8% for men) and inpatient/semi-inpatient nursing (19% vs. 8% for men).

## 4 Discussion

OA represents a growing public health and economic challenge. This study provides the first long-term analysis of direct OA-related healthcare expenditures in Germany using official national data. Between 2002 and 2020, these costs increased by nearly 65%, reaching €12.1 billion in 2020. For comparison, in 2020 diabetes mellitus (ICD-10: E10-E14) accounted for €7.4 billion, depressive disorders (F32-F34) for €9.5 billion, and cardiac insufficiency (I50) for €7.4 billion in direct disease-related healthcare costs in Germany (10). This sharp rise of OA costs reflects demographic aging but also points to limitations in the effectiveness and structure of current OA care strategies.

Musculoskeletal and connective tissue disorders rank among the five most cost-intensive ICD-10 diagnostic groups in Germany based on direct healthcare expenditures, with OA accounting for the highest direct disease-specific costs in this group. This reflects not only the clinical significance of OA, but also its economic impact on the German healthcare system. The majority of total direct osteoarthritis-related costs are attributable to inpatient care facilities, with a significant cost increase observed between 2015 and 2020. Hospital-related expenditures contributed to this trend, even though OA hospitalization numbers increased only slightly between 2015 (416,008 cases), 2018 (418,272 cases), and 2019 (425,763 cases), while the average length of hospital stay for OA declined (2015, 9.8 days, 2019, 8.7 days) (11). This may indicate a higher proportion of patients with complex needs requiring more resource-intensive care within hospitals. A substantial post-pandemic rebound followed, with 470,644 OA hospital admissions recorded in 2023 (11), which will need to be evaluated in future disease cost analyses that include OA-related expenditures beyond 2020. Notably, observed expenditures for inpatient and semi-inpatient nursing care surged by 98%, emerging as the primary driver of the observed cost increase in inpatient and semi-inpatient settings between 2015 and 2020. Additionally, more than half of OA-related outpatient expenditures were attributable to nursing services, which rose by 85% over the same period. Even when accounting for structural policy reforms – such as the 2019 Nursing Staff Strengthening Act (Pflegepersonal-Stärkungsgesetz, PpSG), which likely contributed to rising nursing care costs through mandatory staffing improvements and wage adjustments – the magnitude of the increase indicates a growing demand for long-term care (12), particularly challenging in an aging society. The pronounced rise in OA-related expenditures among adults aged ≥65, and particularly in those aged ≥85, between 2015 and 2020 – especially with regard to OA-related nursing costs – is likely multifactorial. Demographic ageing in Germany has increased the absolute number of older OA patients, as evidenced by rising number of geriatric hospitalized OA patients (10) who often present with greater multimorbidity, frailty, and functional impairment. These factors not only elevate the intensity and complexity of care, but also extend recovery times, resulting in higher cumulative costs. Moreover, multimorbidity in older patients can constitute a contraindication to joint arthroplasty, further leading to a higher reliance on conservative management, geriatric rehabilitation, and long-term nursing care – all of which have experienced increasing unit costs. Rising expectations for mobility and quality of life in older adults may further contribute to rising demand for OA-related medical and rehabilitative services. Taken together, the observed increase in nursing expenditures may, at least in part, reflect the limited effectiveness of current treatment strategies in preventing care dependency, underscoring the urgent need for novel, personalized, and interdisciplinary approaches to OA care. Thus, OA is not only a growing economic burden but also a pressing challenge for long-term care systems, especially in light of the ongoing shortage of qualified nursing staff in Germany (13).

In contrast, costs for pharmacies and physicians declined between 2015 and 2020. This may reflect increased use of generics (14), and concurrent shift toward non-medical services, with greater emphasis on rehabilitation and prevention, as suggested by previous studies (15). Postoperative recovery may also last longer in older patients, further contributing to these costs (16).

Furthermore, findings from this study reveal marked sex-specific differences in OA-related cost distribution across care sectors, with women accounting for 70% of total costs in 2020 associated with higher OA prevalence in women. The 12-month prevalence of osteoarthritis in Germany was determined in 2014/15 to be 21.8% for women and 13.9% for men, irrespective of age (10). Moreover, the higher prevalence of osteoarthritis in women compared to men within the geriatric OA population, reflected in the higher hospitalization rates of women aged ≥65 and ≥85 with OA compared to men, likely contributes to comparatively higher nursing care expenditures among female patients (10). In addition, differences in OA pathophysiology, sex-related health-seeking behavior, and patterns of care utilization between sexes may contribute to the observed cost variations across different health care sectors. However, these explanations remain speculative, as the data of this study do not allow a clear causal attribution of the incurred costs. Nonetheless, our findings underscore the importance of incorporating gender-sensitive approaches in osteoarthritis research, therapeutic development, and healthcare planning.

The economic burden of direct osteoarthritis-related healthcare costs is further exacerbated by its substantial indirect costs, primarily due to disability, productivity losses and early retirement (17). OA related indirect costs due incapacity for work, disability, and mortality were lastly reported for osteoarthritis in the national disease cost calculation in 2008 and therefore not further analyzed in this study. However, as early as 2008, in Germany osteoarthritis accounted already for the loss of 39 out of 1,000 potential work years due to temporary work disability and an additional 30 work years due to permanent disability (18).

Germany’s direct medical costs for OA, as analyzed in this study, are substantial even in an international comparison. Other European countries report lower absolute expenditures. A recent Italian analysis estimated roughly €2.5 billion annual direct OA costs for ~3.9 million OA patients (19). Based on the total number of OA patients, direct healthcare costs in 2017 amounted to about €1.04 billion in Sweden and about 0.72 billion in Norway (20). Direct cost data from the UK indicate that, even as early as 2012, OA-related interventions accounted for £896 million annually (€1.10 billion, exchange rate 2012: £1 ≈ €1.23) (21). In 2015, total U. S. OA related costs were estimated at $193.9 billion (€174.51 billion, exchange rate 2015: 1 US$ ≈ €0.90), based on a 10.5% prevalence of affected individuals in the population (22). Recent data from Australia show direct OA costs in 2015 estimated at AU$2.1 billion reflecting its smaller population, with a forecast for 2030 exceeding AU$2.9 billion (€1.43 billion and €1.97 billion, exchange rate 2015 of 1 AU$ ≈ €0.68) (23). Differences in OA-related cost estimates across countries likely reflect a combination of factors, including variations in healthcare system structures, reimbursement mechanisms, coding practices, cost accounting methods, and population demographics. In Germany, the comparatively high expenditures may be partly attributed to the structural predominance of inpatient care. With nearly twice as many hospital beds per capita as the OECD average and consistently high hospitalization rates – particularly among older adults – Germany exhibits a systemic tendency toward inpatient care (24, 25). Across different health systems – whether in Europe, North America, or Australia – OA poses a consistently heavy economic burden, both in absolute costs and as a proportion of national health expenditures. This international perspective further highlights that urgent strategies are needed to mitigate the increasing burden of OA, a challenge further exacerbated by the still insufficiently understood pathogenesis, which hampers the development of causal therapies and limits current treatment options to symptomatic relief.

Biochemical inflammatory and molecular processes play a critical role in the onset and progression of osteoarthritis (26, 27). A causal pharmacological therapy targeting these OA underlying mechanisms could reduce osteoarthritis risk in aging populations and lower mid and long-term healthcare costs compared to symptomatic treatments. Until such therapies with disease-modifying osteoarthritis drugs (DMOADs) become available, multidisciplinary prevention and care strategies remain essential. Structured physical activity, multimodal pain management, weight control, and adherence to a Mediterranean diet show proven benefits (28–30). Given the heterogeneous nature of osteoarthritis, patient- specific interventions are required.

Despite guideline recommendations, physiotherapy remains underused. Expanded access to qualified physiotherapists and structured self-management programs could help reduce care dependency. Mediterranean dietary pattern exerts beneficial effects on osteoarthritis incidence and symptom severity, which is only partially explained by its impact on BMI (28, 30, 31), highlighting its potential role in both prevention and treatment. Thus, integrating dietary counseling and nutritional support into OA management – potentially via digital or group-based formats – may offer scalable preventive benefits. Additionally, sex-specific research and care planning could enable optimized and gender-sensitive OA treatment strategies, potentially reducing the disproportionate burden observed among women.

Public awareness campaigns and caregiver education could further delay loss of function in high-risk OA patients. Digital health applications (DiGAs) may serve as valuable tools for patient engagement, monitoring, and continuity of care (32), particularly when embedded in reimbursed care pathways. Embedding conservative OA treatments into structured care models – including home care, rehabilitation, and nursing home settings – could enhance tertiary prevention by maintaining mobility and autonomy. Implementing these approaches will require adjusted reimbursement models that strengthen incentives for outpatient prevention and improve compensation in geriatric care – in order to counteract the nursing staff shortage. We propose the establishment of specialized OA centers that integrate medical, rehabilitative, nutritional, and digital services to deliver individualized, multidisciplinary care across sectors. To strengthen long-term system integration, we further recommend evaluating the potential of embedding OA-specific care strategies into national chronic disease management frameworks. Although osteoarthritis is not currently included in Germany’s Disease Management Programs (DMPs), our findings suggest that its inclusion could be beneficial in light of the disease’s growing societal and economic burden.

Several limitations must be considered when interpreting the findings of this study. However, these limitations do not compromise the study’s central findings. This study relies on data from the German Federal Statistical Office, which uses a top-down cost-of-illness approach. Since the dataset consists of administrative, aggregated healthcare cost estimates without access to individual-level data, statistical analyses such as confidence intervals or *p*-values could not be calculated. Consequently, while the observed differences and trends in OA-related costs across time, age groups, and sexes appear substantial, they should be interpreted descriptively. Disease cost data are only published for selected reporting years (2002, 2004, 2006, 2008, 2015, and 2020). A more detailed year-by-year trend analysis was therefore not feasible and was deliberately avoided to maintain methodological accuracy. As of July 2025, no updated disease cost data beyond 2020 are available. This limits long-term projections but does not affect the internal consistency of the current analysis. Our analysis is based on aggregate OA-related costs, which reflect both disease-specific care demands and broader systemic cost drivers; the dataset does not permit a clear causal disentanglement of these factors. Although preliminary dataset evaluations ensured internal comparability across years, methodological inconsistencies – particularly in the recording of expenditures for “offices of physicians” and “pharmacies” – may have introduced bias when comparing data across time points. The annually adjusted DRG system remained largely unchanged between 2015 and 2020, suggesting a minimal impact on cost development. In contrast, the annual adjustment of regional and national base case values, which increased by 3.8% in 2020 compared to the previous year (33), represents a relevant cost driver. Finally, potential confounding effects from the COVID-19 pandemic must be considered, although the disease cost database reflects primary diagnoses only, while COVID-19 was classified as a secondary diagnosis according to WHO coding guideline. Nevertheless, pandemic-related effects, such as the reduction in osteoarthritis treatment cases in 2020, may have influenced costs and triggered a compensatory post-pandemic increase. This requires further evaluation in subsequent disease cost assessments.

In conclusion, this study demonstrates that osteoarthritis represents a major and escalating economic challenge for the German healthcare system, driven primarily by demographic aging and the increasing demand for inpatient and nursing care services. Future healthcare planning must prioritize and support the implementation of OA-specific prevention strategies, gender-sensitive and personalized conservative treatments, and better integration of OA management into geriatric, rehabilitative, and outpatient care frameworks. Embedding OA care into structured, community-based programs and leveraging digital health technologies could help reducing long-term care dependency and improve cost-efficiency. To ensure the sustainability of healthcare systems in aging societies, OA must be addressed not merely as a clinical condition, but as a structural challenge – requiring integrated, data-driven, and forward-looking public health strategies.

## Data Availability

The original contributions presented in the study are included in the article/supplementary material, further inquiries can be directed to the corresponding author.
